# Preemptive endoluminal vacuum therapy with the VACStent—A pilot study to reduce anastomotic leakage after Ivor Lewis hybrid esophagectomy

**DOI:** 10.3389/fsurg.2023.1133083

**Published:** 2023-03-28

**Authors:** Jonas Lange, Claus Ferdinand Eisenberger, Judith Knievel, Anne Linderer, Markus Maria Heiss

**Affiliations:** Department of Abdominal, Tumor, Transplant and Vascular Surgery, Cologne-Merheim Medical Center, Witten/Herdecke University, Cologne, Germany

**Keywords:** endoscopic treatment by vacuum therapy (EVT), upper gastrointestinal wall defects, anastomotic leakage (AL) after esophagectomy, VACStent, preemptive therapy

## Abstract

**Introduction:**

Endoscopic treatment by vacuum therapy (EVT) or covered stents has emerged as an improved treatment option for upper gastrointestinal wall defects and is regarded as an improved treatment option for anastomotic leakage (AL) after esophagectomy. However, endoluminal EVT devices may lead to obstruction of the GI tract; and a high rate of migration and missing functional drainage has been shown for covered stents. The recently developed VACStent, a combination of a fully covered stent within a polyurethane sponge cylinder may overcome these issues allowing EVT while stent passage is still open. Initial clinical applications have demonstrated efficacy, practicability and safety in the treatment of esophageal leaks (AL).

**Methods:**

In this pilot study, 9 patients with high-risk anastomosis after neoadjuvant therapy undergoing hybrid esophagectomy received the VACStent in a preemptive setting for the assessment of the reduction of the AL rate, postoperative morbidity and mortality.

**Results:**

Technical success of the application of the VACStent® was achieved in all interventions. One patient experienced anastomotic leakage 10 days after esophagectomy and was successfully treated with two consecutive VACStents and a VAC Sponge. In summary, mortality in-hospital was 0% and anastomotic healing was uneventful without septic episodes. No severe device-related adverse events (SADE) nor significant local bleeding or erosion could be observed. Oral intake of liquids or food was documented in all patients. The device handling was regarded uncomplicated.

**Discussion:**

The preemptive application of the VACStent offers a promising new option for improved clinical treatment avoiding of critical situations in hybrid esophagectomy, which should be validated in a large clinical study.

## Introduction

Vacuum therapy was applied initially for infected and ischemic wounds (1) and later on adopted as endoluminal vacuum therapy from surgical endoscopists as treatment for anastomotic leakage (AL) after rectal surgery in the lower gastrointestinal (GI) tract (2) as well as AL after esophago-gastrectomy (3).

The risk of anastomotic insufficiency in resection surgery of the upper GI tract has remained high in recent years. Modern studies show an insufficiency rate of up to 15% (4).

During Endoscopic treatment by vacuum therapy (EVT) the wound compartment is treated by a negative atmosphere pressure requiring a suction pump and an airtight seal. The negative pressure causes attachment of the surrounding tissue creating a closed negative pressure environment. Evacuation of secretions, removal of wound debris, containment of the defect, reduction of the interstitial edema as well as increased oxygen saturation and promotion of tissue granulation as well as microcirculation are beneficial effects of this technique depending on the intensity of the applied negative pressure (5–8).

Various devices have been developed for indications in- or outside the intestinal lumen and in combination with surgical, radiological, and other endoscopic interventions.

However, the only approved medical device is the Eso-SPONGE® (B. Braun Melsungen AG, Germany), a macroporous polyurethane (PU) sponge system combined with a pressure-resistant plastic suction tube. It is able to build up suction endoluminally in the area of the leakage and thus drain and close the wound. The disadvantage is that the upper gastrointestinal tract is blocked and no early postoperative food build-up is possible. Based on the good clinical experience to date, the concept of prophylactic sponge application directly intraoperatively after creation of the anastomosis was developed (9). On the one hand, this should reduce the frequency of insufficiencies and, on the other hand if an AL happend, completely prevent the development of a septic focus. Clinical experience to date shows that both goals can indeed be achieved (10, 11). However, the evidence to support these hypotheses is still lacking.

The recently developed VACStent® (VACStent GmbH, Fulda) consists of a combination of PU foam with a covered SEMS in a manufactured setup and is suitable for intraluminal EVT due to the cylindrical shape of the PU foam (12, 13). Initial clinical applications have demonstrated that the VACStent was easy to insert and able to seal off esophageal leakage and anastomotic failure while still enabeling the passage for liquids and mashed food. Considering the high incidence and deleterious effects of AL, we implemented preemptive EVT (pEVT) in patients undergoing esophagectomy and gastric tube reconstruction.

## Patients and methods

### The VACStent

The VACStent comprises a self-expanding nitinol stent covered with a silicone-membrane impermeable to liquid and gas (VACStent GmbH, Fulda). Affixed to the exterior of the stent is a polyurethane sponge cylinder (thickness 10 mm) connected to an external vacuum pump *via* a fixed small gauge (12 F) catheter.

After application of negative pressure the flanged ends of the stent are in contact with the intestinal wall, sealing the sponge cylinder against the intestinal fluids. The constant suction induces an immobilization of the VACStent on the intestinal wall preventing stent-migration.

The VACStent is loaded on a flexible introducer system, which is inserted transorally in over-the-wire technique. The positioning can be controlled by fluoroscopy and the unfolding of the stent can easily be followed on screen. Alternativly the deployment of the VACStent can also be observed by a small Endoscope placed in parallel to the introducer system. After release, the nitinol filaments unfold the VACStent to its original size. The repositioned transnasal suction catheter is connected to a vacuum pump with a negative pressure ranging of −80 mmHg.

### Study design

This pilot study was a single-center, open-label analysis of prospectively collected data from patients selected from a register of the new VACStent system [VAC-Stent registry: (https://clinicaltrials.gov/ct2/show/NCT04884334)]. The study protocol was approved of the Institutional Review Board of the Witten/Herdecke University (Nr. 34/2020, 30.04.2020). The main focus of the investigation was the efficacy, safety, applicability and migration resistance of the VACStent, as well as the unrestrained passage of swallowed liquids through the stent. The VACStent treatment was performed by experienced endoscopists at one German tertiary center (Klinikum Köln-Merheim).

### Patient collective

From a prospectively maintained database, we identified 9 patients who underwent esophagectomy with intraoperative pEVT between November 2021 and June 2022 in our department.

Records of patients were reviewed with respect to demographic characteristics, oncological parameters, surgical procedures, and the postoperative course up to 12 months after surgery.

### Study endpoints

The primary endpoints of this study were the intraoperative safe technical practicality of the VACStent and its successful coverage of the esophagus anastomosis. Secondary endpoints were postoperative mortality, morbidity and AL rate, defined according to the Esophageal Complications Consensus Group (ECCG) (14).

The endpoint was achieved in full if the VACStent could be deployed and continuous suction *via* the sponge-cylinder applied. This was assessed by endoscopy during the procedure and continuous control of the established negative pressure. Further objectives were the assessment of septic signs and symptoms, and complications, in particular VACStent dislocation, erosion of tissue within the wound cavity or at the bowel wall, also bleeding or ulcer in the wound cavity or at the VACStent site.

Eligible was any patient with a surgical procedure, and that the anastomosis was reachable by the applicator-system of the VACStent, provided that informed consent had been given.

Excluded were patients without accessibility of the VACStent, a cervical anastomosis and patients needing full anticoagulation or with thrombocytopenia <20.000/µl.

### Surgical technique and perioperative management

The surgical procedures were hybrid (laparoscopic/mini-thoracotomy) Ivor Lewis esophagectomy with high intrathoracic circular stapled end-to-side esophagogastrostomy. Gastric tube formation and dissection of the distal esophagus was done laparoscopically in all but one cases. There a median laparotomy was performed due to prior surgeries.

Access to the right thoracic cavity was done by mini-thoracotomy in the 5th ICR in all cases (incision length 8–12 cm). Resection of the v. azygos was done in all cases. Esophagogastric anastomosis was done with a circular stapler (25–29 mm). Then the VACStent was applied intraopertively and the correct positioning was under direct digital control by the surgeon.

Postoperative oral nutrition with water/tea started at the 1st postop day and was extended the following days to high-caloric liquids (Fresenius, Bad Homburg). It was recommended to leave the VACStent in place for at least 5 days. Then a transoral endoscopy was performed under sedation (propofol) and the VACStent removed. If an AL should be detected a new VACStent should be delivered and again renewed until the AL has healed.

Antibiotic prophylaxis (Tazobac, Pfizer PFE GmbH, Germany) was given routinely for 5–7 days.

### VACStent application

After performance of transoral endoscopy a stiff guide wire was placed under direct vision in the gastric-tube or duodenum, then the delivery system carefully advanced over the wire and the VACStent deployment observed *via* a small 8 mm endoscope, which paralleled the delivery system. The application system and guidewire were then removed and the suction catheter passed retrograde through the nose. Before connecting the suction catheter to a VAC-pump (e.g., Curasul®, BSN medical GmbH, Hamburg, Germany) with a plastic Y-adapter, retrograde rinsing with 0.9% NaCl solution of the sponge-cylinder was performed to facilitate and ensure the deployment of the open-cell PUR-sponge. The continuous suction pressure was −80 mmHG in all cases.

The protocol recommends the length of stay for a preemptive VACStent of at least 5 days.

Before removal of the VACStent extensive retrograde rinsing of the sponge *via* the drainage-tube (at least 40 ml 0.9% NaCl) was recommended and vacuum suction should be stopped for at least 2 h before VACStent removal. Removal was performed endoscopically with forceps to pull at the retrieval loops placed at the ends of the VACStent.

### Data collection and analysis

Safety, efficacy, and clinical course of the VACStent treatment were analyzed daily from patient enrollment until hospital discharge and during follow-up visits until 12 months post-op. In 8 of 9 patients follow-up endoscopy for long-term data of the VACStent treatment was performed. All data were collected in a CRF, entered in a database, and analyzed.

## Results

9 patients have been enrolled in one site in Germany (Cologne-Merheim) between November 2021 and June 2022, 7 male and two female. All patients underwent subtotal resection of the tumor-bearing esophagus trans-thoracically with concomitant lymphadenectomy. En-bloc removal of the upper third of the stomach together with the small curvature and lymphadenectomy was also performed. Except for one patient with a microscopic tumor remnant in the esophageal incision margin, the tumor could be completely removed (R0) in 8 patients. Reconstruction was performed in all 9 patients according to Ivor-Lewis with an end-to-side esophago-gastrostomy.

In total 11 VACStents were placed endoscopically in 9 patients. Placement was reported to be easy or only moderately difficult. In 10 cases correct positioning and deployment of the VACStent was technically successful, only one repositioning (Endoscopic repositioning with grasping forceps in case of intraoperative too deep stent release directly in the course of primary placement of the stent) was necessary. The endpoints technical practicality of the VACStent and its successful coverage of the esophagus anastomosis were met in all patients. The average stent indwelling time was 5.7 days (range 4–7 days). One patient developed an esophagus leakage after 10 days which was treated successfully with two further VACStent applications and a PU foam for 14 days. Complete morphological healing of the anastomosis was seen in all patients (100%). The median hospital stay was 14 days (range 12–29) and the median ICU stay was 3 days (range 1–9).

No patient experienced sepsis, clinical pneumonia or aspiration pneumonia, severe dyspnoe or death.

Oral intake of water and liquids was possible in all patients. In 7 of 9 patients additional dispersed oral food was swallowed. In no case a severe adverse device associated event (SADE) was reported or a VACStent migration or dislocation observed. No clinically significant erosion, perforation or ulcer was noted and also no local bleeding, neither throughout the VACStent site nor in the wound cavity. Also, no significant malfunctions of the drainage capacity of the VACStent were reported. Removal of the 11 VACStents was performed without major problems.

Later Follow-up was done endoscopically in 8 of 9 patients and revealed no anastomotic problem or stenosis after median 5 months (range 2–12 months) (Tables 1–3).

**Table 1:** Patient characteristics (*n* = 9).

Age, years (mean ±SD, range)	63.7 ± 6.8 (55-78)
BMI, kg/m^2^ (mean ±SD, range)	24.6 ± 6.5 (15.5-33.95)
Male; female, n	7; 2
ASA status, n (%)	
Grade II	1 (11)
Grade III	8 (89)
Grade IV	0 (0)
Histology, n (%)	
AC	7 (78)
SCC	2 (22)
Benign	0 (0)
Tumor location, n (%)*	
Proximal half of esophagus	2 (22)
Distal half of esophagus	5 (56)
Esophagogastric junction (Siewert II)	2 (22)
Preoperative therapy, n (%)*	
None	0 (0)
Radiochemotherapy	3 (33)
Chemotherapy	6 (67)
UICC stages, n (%)*	
I	2 (22)
Ia	1 (11)
Ib	0 (0)
II	0 (0)
IIIa	1 (11)
IIIb	3 (33)
IVa	1 (11)
IVb	1 (11)

IQR, interquartile range; BMI, body mass index; ASA, American Society of Anesthesiologists; AC, adenocarcinoma; SCC, squamous cell carcinoma; UICC, Union Internationale Contre le Cancer TNM Classification for Esophageal Cancer 8th edition.

^a^ Patients with malignant indication *n* = 9.

**Table 2:** Surgery characteristics.

Surgical approach	
Laparoscopic/mini-thoracotomy Ivor Lewis	8
Open laparotomy	1
OP-length (Minutes, median, IQR)	293 (277-394)
Blood-loss (ml, median, IQR)	800 (500-1100)
Localization of anastomosis	25 (25-30)
(cm from teeth row, median, IQR)	

**Table 3:** Endpoints.

Primary endpoints	n (%)
Intraoperative safe technical practicality of the VACStent	9 (100)*
Successful coverage of the esophagus anastomosis	9 (100)**
Secondary endpoints	
Postoperative mortality	0 (0)
Postoperative morbidity- AL rate	1 (11)***
Oral liquid food uptake	9 (100)
Further objectives	
Assessment of septic signs and symptoms	0 (0)
VACStent dislocation	0 (0)
Erosion of tissue at the anastomosis or at the bowel wall	0 (0)
Bleeding or ulcer at the anastomosis or at the VACStent site	0 (0)****

Defined according to the Esophageal Complications Consensus Group (ECCG) (14).

^a^ The endpoint was achieved in full if the VACStent could be deployed and continuous suction *via* the sponge-cylinder applied.

^b^ This was assessed by endoscopy during the procedure and continuous control of the established negative pressure.

^c^ 10 days after second treatment with VACStent closure of the AL and granulation.

^d^ 5 patients with reflux esophagitis.

## Discussion

The idea of this study started with the observation, that immediate application of pEVT in fresh endoscopic or surgical lesions, or in spontaneous esophageal rupture (Boerhaave syndrome) may result in accelerated healing (12). The increased anastomotic blood flow, modulation of cytokines, enhanced angiogenesis with deposition of granulation are known mode of action (MoA) of EVT and may support the sealing of micro-anastomotic defects at a very early stage even in a preemptive setting after esophagectomy. These observations led to the concept of prophylactic intraoperative EVT after esophagectomy exhibiting convincing initial clinical data for the Eso-SPONGE® in the prevention of anastomotic suture line failure (15). The clinical outcome of a preemptive clinical study with the Eso-SPONGE® (16) was promising with a zero 30-day mortality and an AL rate of 5% without septic complications.

These promising results led to the concept of the preemptive EVT with the VACStent. The advantage of the VACStent technology is avoidance of sponge obstruction. Early oral liquid and food uptake was possible for all patients, a major benefit of the VACStent design principle enabling a free passage through the VACStent body. This observation is contradictory to a previous study of the VACStent for the treatment of esophageal leaks (13). The manufacturer developed an improved VACStent model, used in this pilot study, which did not exhibit a luminal narrowing due to longitudinal fold formation in the covering.

The hypothesis of this pilot study was to verify whether the concept of intraoperative EVT can be achieved with the VACStent. However, not only a potential promotion of wound healing but also a possible impairment of anastomotic healing should be recorded. This is because direct intraoperative application could well be associated with mechanical stress or impairment of the fresh anastomosis. This could not be detected in any patient, neither in the area of the circular staple suture, at the blind closure of the gastric tube nor at the mucosa of esophagus or stomach. Intraoperative application was simple and without its own previously unknown problems or complication. Precise placement is even simplified by the surgeon's digital control.

There are no accepted standards for ideal treatment duration and optimal negative system pressure with EVT. However, complete removal of the VACStent without residua was unproblematic and an average negative pressure of 80 mmHg was sufficient to promote formation of granulation tissue.

The treatment with the VACStent should be carefully counterbalanced against its potential risks. Esophageal stenosis with clinical dysphagia might be possibly triggered by EVT (17). Late complications of pEVT like anastomotic strictures may have been missed in this pilot study and should be evaluated in future prospective clinical trials.

Implantation of the VACStent results in increased therapy material costs. In our center the endoscopy for implantation in the operating room after the anastomosis and the control gastroscopy after 5–7 days are performed as standard, so that an increased resource expenditure does not arise. The examination times for these endoscopies are minimally extended. The prevention of anastomotic insufficiencies and the faster convalescence of the patients postoperatively compensate for a large part of the additional costs.

A significant advantage of this pEVT with the VACStent is the early anastomotic assessment after 5–7 days and if morphological AL signs appear the further treatment, thus avoiding the development of clinical sepsis conditions. Even if ischemic loss of the tip of the gastric tube were to occur, this would be treatable as long as the ischemic zone is covered by the sponge cylinder of the VACStent. A problem not captured by pEVT is late AL, as seen in the patient in the study with AL on 10 post op day, 3 days after removal of the VACStent during an inconspicuous endoscopy.

Overall, all patients in the pilot study were high-risk patients with significant prior disease and, most importantly, neoadjuvant chemo and radiochemo therapies. Even if the pilot study cannot say anything about the expected efficacy, these good results give hope that a real improvement in healing can be achieved. A clinical situation that can then also be transferred to other problematic anastomoses such as in bariatric surgery or other difficult esophageal-gastric anastomoses.

## Conclusion

In this single-center feasibility study, the applicability and efficacy of the VACStent in the preemptive management of endoscopical esophageal resection could be demonstrated.

In all patients, the VACStent treatment was performed without any significant problems. Morphologic healing was observed in all patients after an average of 5.7 days of VACStent placement. One patient was treated three times with a VACStent due to an anastomotic leakage which healed completely after 2 weeks of the second intervention.

Insertion and release as well as the removal of the VACStent were easy or moderately difficult and did not differ significantly from conventional stent systems.

Severe VACStent-associated complications (SADE reports) did not occur in any of the 11 treatments. Reposition of the VACStent occurred in only one case. No dislocation and no migration were observed during the later course of treatment.

Preemptive endoluminal vacuum therapy with the VACStent is a safe procedure that may reduce AL formation and related morbidity by promoting primary anastomotic healing. Preemptive endoluminal vacuum therapy may be particularly valuable in patients with relevant comorbidities and increased risk for AL.

VACStent is a safe and feasible endoscopic treatment option for leaks of the upper gastrointestinal tract. The VACStent, designed to be capable of combining the benefits of EVT with those of stenting while being simple and safe to apply, may allow immediate wound closure and effective drainage of endoluminal wounds by preemptive application. The efficacy of preemptive VACStent application needs to be validated in an extensive clinical study.

## Data Availability

The raw data supporting the conclusions of this article will be made available by the authors, without undue reservation.
